# Complete sequencing of the chloroplast genomes of two *Medicago* species

**DOI:** 10.1080/23802359.2017.1325336

**Published:** 2017-05-25

**Authors:** Yan Weihong, Shi Wengui, Liu Lei, Ma Yubao, Chen Libo, Wang Zhaolan, Hou Xiangyang

**Affiliations:** Ministry of Agriculture, Institute of Grassland Research of CAAS/Key Laboratory of Grassland Resources and Utilization, Inner Mongolia, Hohhot, China

**Keywords:** Medicago, chloroplast genome, phylogenomic analysis

## Abstract

In this study, we aimed to determine the complete nucleotide sequences of the chloroplast genomes of *Medicago sativa* L. and *Medicago falcata* L. and perform their phylogenomic analysis. The chloroplast genomes of *M. Sativa* and *M. falcata* were found to be 125,095 and 125,810 bp in length, respectively. Additionally, the AT contents of *M. Sativa* and *M. falcata* are 66.1% and 66.2%, respectively. Each chloroplast genome contained the same 109 unique genes, including 29 transfer RNA genes, 4 ribosomal RNA genes, and 76 protein-coding genes. Phylogenomic analysis based on complete chloroplast genomes from the *Medicago* showed that the geographical environment has no significant effect on their genomic composition.

*Medicago* plants are one of the most widely cultivated perennial forage legumes worldwide. The plant materials originating in different geographical, ecological, and climatic regions were used in this study. *Medicago sativa* seeds were obtained from Inner Mongolia, China (42°02′N,111°35′E), and *M. falcata* seeds from Khovd, Mongolia (46°49′N,91°45′E). The seeds were not deposited into the National Library and were cultivated and spread planting at Taipusiqi test sites, the Institute of Grassland, Chinese Academy of Agricultural Sciences (Inner Mongolia, China). After allowing the plants to grow for 12 weeks in a greenhouse at 22 °C with a photoperiod of 16 h, their leaves were collected for further experiments.

Total genomic DNA was extracted from 150 mg of actively growing fresh leaves using a modified CTAB method (Doyle 1987). Nine novel universal primer pairs (Yang et al. 2014) were used to amplify *Medicago* chloroplast genomes. Long-range PCR method was performed to amplify target fragments, and purified DNA was used to construct short-insert (350–550 bp) libraries. Solexa high-throughput sequencing system (Illumina Hiseq2500, San Diego, CA) was used to generate raw sequence reads. DNA from each individual was indexed using tags and pooled together for sequencing at Beijing Yuanquanyike Biological Technology Co., Ltd (Beijing, China).

*Medicago sativa* and *M. falcata* were sequenced individually to produce 8,325,635 and 7,689,970 clean paired-end reads, respectively, with 96 bp in average length. The amplified fragments were sequenced by Sanger-sequencing using primers designed on the basis of the reference genome. First, various quality control checks on the short reads were performed using solexa QA to filter the Illumina data for high-quality and vector- and adaptor-free reads (Cox et al. 2010). High-quality short reads were assembled into contigs using SOAP denovo (Luo et al. 2012). Subsequently, we identified highly similar genome sequences by Blast with the default search parameters. The GapCloser software (University of Hong Kong, Pokfulam, Hong Kong) was used to fill the gaps with high-quality short sequences, and then the gaps were verified by Sanger sequencing. Finally, to avoid assembly errors, some pairs of primers were designed for PCR amplifications. The final complete chloroplast genome sequences were deposited into GenBank with accession numbers of KU321071 (*M. sativa*) and KX831887 (*M. falcata*).

In order to examine the phylogenetic utility of different regions, phylogenetic analyses were performed based on two data partitions (complete cpDNA sequences and 72 protein-coding exons) from five Leguminosae plants’ cp genomes to construct a Maximum-Likelihood phylogenetic tree. Phylogenetic relationships with bootstrap values and posterior probabilities are presented in Figure 1. Phylogeny among the two newly sequenced *Medicago* chloroplasts and other chloroplasts was constructed on the basis of the genome sequences and minor allele frequencies (MAFs) in the polymorphism sites. As clear differences were observed between chloroplast and the newly sequenced chloroplasts, thaliana chloroplast was used as an outgroup directly.

**Figure 1.**
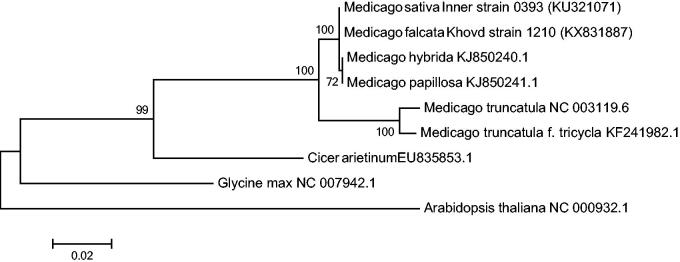
ML phylogenetic tree is rooted with *Arabidopsis thaliana*. The most basal lineage was *Glycine max* (L.) *Merr.* followed by the *Cicer arietinum L.* The next branch included *Medicago*, of which *M. sativa* and *M. falcata* showed higher similarity.

We expect the two complete Medicago chloroplast genome sequences and the genomic variations might provide some valid information for further studies on the genetic relationship of this genus.
